# White Spot Lesions in Fixed Orthodontics: A Literature Review on Etiology, Prevention, and Treatment

**DOI:** 10.7759/cureus.65679

**Published:** 2024-07-29

**Authors:** Deem Al-Blaihed, Omar El Meligy, Khlood Baghlaf, Rabab A Aljawi, Shahad Abudawood

**Affiliations:** 1 Dentistry, Ministry of Health, Jeddah, SAU; 2 Pediatric Dentistry, King Abdulaziz University Faculty of Dentistry, Jeddah, SAU; 3 Pediatric Dentistry and Dental Public Health, Alexandria University, Alexandria, EGY; 4 Pediatric Dentistry, King Abdulaziz University, Jeddah, SAU

**Keywords:** “camouflage”, “preventive measures”, “post-orthodontic white spot lesion”, “enamel remineralization”, “enamel demineralization”, “decalcification“, “fixed orthodontic treatment”

## Abstract

White spot lesions (WSLs) are a common complication after treatment using fixed orthodontic appliances. Decalcification of enamel surrounding fixed orthodontic appliances, known as WSLs, poses a significant aesthetic difficulty during and after treatment, as the purpose of fixed orthodontic therapy is to improve facial and dental appearance. Modern dentistry utilizes remineralization therapies to non-invasively treat WSLs to prevent the progression of disease and enhance the strength, appearance, and functionality of the affected tooth. This review aims to identify and assess the etiology, formation, and risk factors, as well as current treatment approaches used in achieving WSLs remineralization, induced by fixed orthodontic appliances. An electronic search on the PubMed and ScienceDirect databases was performed using a selected keyword. A total of 172 studies (from 2013 to 2023) were retrieved. Their references were also checked to find other relevant studies. Duplicate copies were excluded. After the abstract and full-text screening, only 39 studies were included. Even though numerous studies address the different treatment modalities for managing post-orthodontic WSLs, such as antiseptics; fluorides such as dentifrices, mouthwash, and varnish, and remineralizing agents such as casein phosphopeptides amorphous calcium phosphate*, *biomimetic self-assembling peptides, lasers, bleaching, microabrasion, and resin infiltration. There is a lack of evidence-based studies that examine the long-term effects of WSL treatment. Further well-performed controlled clinical trials with long-term follow-up are needed to establish best clinical practice.

## Introduction and background

Decalcification or demineralization refers to the loss of calcified tooth substance [1]. Enamel demineralization manifests clinically as white spot lesions (WSLs) produced by changes in optical characteristics induced by subsurface mineral loss [2]. Bacteria in tooth plaque produce acid, which causes dissolving of the structure [1]. WSLs are a commonly observed negative consequence of orthodontic therapy, resulting primarily from inadequate oral hygiene [3]. During orthodontic treatment, it has been observed that fixed orthodontic components, such as brackets, have the potential to result in a greater deposition of biofilm [4]. Consequently, individuals who consume a highly cariogenic diet and have an inadequate oral hygiene regimen have a tendency to get WSLs, alternatively referred to as early caries lesions, as they indicate the initial stage of enamel demineralization [4,5].

WSLs imply that there is a subsurface area with most of the mineral loss beneath a relatively intact enamel surface. WSLs are typically in the International Caries Detection and Assessment System (ICDAS) II 1-2 range [6]. The prevalence of WSLs shows a variation across different studies; Boersma et al. reported that WSL exhibit a prevalence of 97% [4], but the findings of a meta-analysis indicate that the prevalence of WSLs was 68.4% and an incidence rate of 45.8%, respectively. This underscores the importance of implementing specific preventive measures to minimize the development of WSLs [3]. These discrepancies might be attributed to the choice of research method and criteria, as well as the inclusion or exclusion of pre-existing enamel lesions [7]. Studies using more sensitive diagnostic procedures than basic visual inspection, such as those employing autofluorescence generated by teeth subjected to high-intensity blue light, quantified light-induced fluorescence (QLF), or the Diagnodent, (DIAGNOdent 2190, KaVo, Biberach, Germany), a device that assesses the degree of demineralization of teeth through fluorescence emitted by laser-scanned teeth, resulted in the highest prevalence [8]. WSLs were found to be two and a half times more frequent in the maxillary teeth in comparison to mandibular teeth [3]. The WSLs were symmetrically distributed between the arches, and the teeth that were most affected teeth were the maxillary laterals, followed by maxillary canines, then mandibular canines [3]. Orthodontic specialists continue to face this issue due to patients' failure to use proper brushing techniques and auxiliary tools [9]. Remineralization therapy has emerged as a popular treatment approach for addressing these lesions [5]. Depending on the type and severity of these lesions, several treatments have been suggested for treatment [10]. Treatments that are advised range from less invasive methods like using fluoride toothpaste at home to more invasive ones like composite restorations [10]. The treatment of white spot lesions has been a long-standing topic of dispute. Despite the urgent need for treatment, this remains a divisive topic.

Aim

The aim of this review is to identify and assess current treatment approaches used in achieving white spot lesion remineralization, induced by fixed orthodontic appliances.

## Review

Methods

Search Strategy

An electronic search was conducted on articles published in the recent 10 years (from 2013 to 2023) that was taken into consideration, using PubMed and ScienceDirect databases. The most relevant articles were chosen by first reading the title, then the abstract, and lastly the entire text. We omitted articles that compared removable orthodontic appliances to fixed orthodontic appliances with ceramic brackets. Research conducted on human teeth received greater attention than those conducted on the teeth of animals. Two researchers reviewed the articles. In case of any discrepancy between the two researchers, a third reviewer handled the discussion to reach an agreement. Several keywords were used to identify the studies for this review, including “fixed orthodontic treatment”, “decalcification”, “enamel demineralization”, “enamel remineralization”, “post-orthodontic white spot lesion”, “preventive measures”, “camouflage” were the terms used in the search.

Eligibility Criteria

The inclusion criteria for the selection of the study included PICO: (1) Participants: WSL formation after treatment with fixed orthodontic therapy. (2) Intervention: Remineralization agents and minimally invasive dental treatments administered to areas that are susceptible to WSL in patients during or after orthodontic treatment. (3) Comparison: Remineralization and minimally invasive dental therapy effect in comparison to baseline or to controlled groups. (4) Outcome indicator: Visual improvement and clinical parameter enhancement of WSLs.

Results

**Figure 1:**
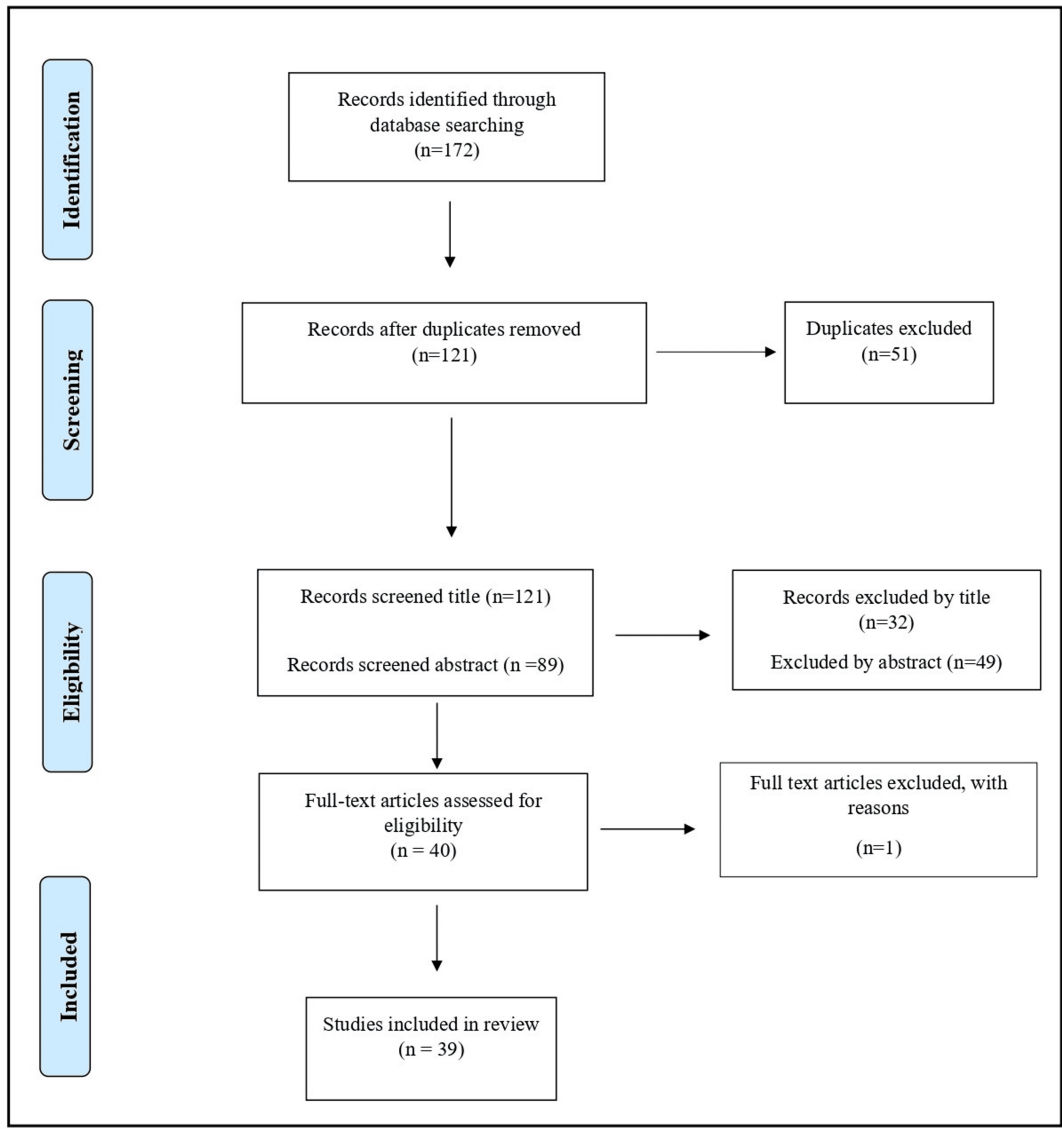
A flow diagram showing the number of articles identified at each stage of the search.

Figure 1 shows the search flowchart - 172 studies were retrieved from PubMed and Science Direct databases. Their references were investigated as well to find other relevant studies. After removing the duplications, a total of 121 studies were screened. In the current literature review, a total of 39 full texts were included in the narrative review.

Discussion

Formation of White Spot Lesions

Fixed orthodontic therapy may provide retentive site-associated dental biofilm accumulation [11]. It was found that patients receiving orthodontic treatment had significantly higher salivary levels of *Streptococcus mutans *(*S. mutans*) [12]. Additionally, changes in dental plaque are seen with orthodontic appliances, such as increased s.mutans, and lactobacillus bacteria, as well as a decreased resting pH of biofilm. Also, excessive material near orthodontic brackets can promote bacterial growth [13]. WSLs, which are classified as incipient non-cavitated decay, are the result of bacterial plaque activity [14], as they are the first clinical evidence of this demineralization [8]. An intact layer is followed by a subsurface porous region referred to as "the body of the lesion" in early lesions. Due to the increased pore volume of these demineralized regions, the refractive index of these lesions is distinct from that of adjacent healthy tooth structure. An increase in lesion porosity corresponds to a greater concentration of air and water, which alters the refractive index [15]. The white spot lesion is composed of four distinct histological layers. The lesion progresses in the following order of layers from its deepest point to its surface: translucent, dark, lesion body, and superficial [16]. Losses of enamel mineralization have been identified in these areas, which manifest clinically as relatively broad, porous, brown, or chalky-white patches that are abrasive to the touch. This phenomenon is associated with the altered diffusion of light in comparison to enamel which is normally mineralized [9].

Etiology and Risk Factors for White Spot Lesions

The host's susceptibility is critical, and it considers both systemic and local factors: the quality and quantity of salivary flow, as well as the enamel's resistance to acid dissolution, are the primary systemic factors, whereas the malposition of teeth and the presence of fixed appliances are the most significant local factors [17]. Within the field of orthodontics, WSLs may be caused by two factors: the prolonged accumulation of plaque on tooth surfaces and the challenges associated with maintaining oral hygiene around bonded teeth. The accumulation of plaque causes a decrease in pH, which in turn upsets the demineralization-remineralization equilibrium in favor of mineral loss, or demineralization. This mineral loss can ultimately result in the development of WSLs and cavitation that extends into the dentin [18]. Additionally, enamel etching performed prior to the placement of fixed orthodontic appliances may have contributed to the increase in caries [19]. Moreover, WSLs prior to orthodontic treatment are considered a risk factor for the development of new lesions [20], with other risk factors including poor oral hygiene, excessive consumption of sugary beverages, frequent intake of fermentable carbohydrates, excessive bonding, decayed or previously treated teeth, and orthodontic therapy treatment period [20, 21]. Also, the type of orthodontic appliance, age, gender, and oral flora were also associated with the formation of WSL [22].

Distribution of White Spot Lesions

WSLs are most commonly found around brackets and on the gingival margin, with the labiogingival area of lateral incisors being the most common [8,10]. The distance between the bracket and the free gingival margin is shorter for lateral incisors, which is also more conducive to the accumulation of plaque [10]. WSLs were found to be two and a half times more frequent in the maxillary teeth in comparison to mandibular teeth. The WSL were symmetrically distributed between the arches, and the teeth that were most affected teeth were the maxillary laterals, followed by maxillary canines, then mandibular canines [3]. Under orthodontic wires, there was a considerable rise in the occurrence of WSLs near the bracket bases or between the brackets and the bonded bands [23], as well as with full coverage rapid maxillary expanders [24].

It was noted that WSL occurrences of full dental arches in participants treated with the lingual WIN (DW Lingual Systems, Bad Essen, Germany) appliance were noticeably lower [25]. White spot lesions develop early and may be noticeable after 4 weeks of orthodontic therapy [26], while other carious lesions take up to 6 months [8]. It was also found that the number of WSLs increased within the first six months of treatment [27].

Prevalence of White Spot Lesions

The WSL decalcifications have been linked to a higher rate of occurrence in people with fixed appliances [28], while males are affected at a higher rate than females [29]. The prevalence of WSL during orthodontic treatment has been shown to range between 2% and 97% across multiple research studies [3, 20]. These variations may be explained by the multiple methods employed to detect them, which include visual inspection, photos, fluorescence approaches, and optical approaches such as DIAGNOdent (DIAGNOdent 2190, KaVo, Biberach, Germany), QLF, and digital image fiberoptic transillumination (DIFOTI) [3]. The greatest rate of prevalence was found in studies utilizing more sensitive diagnostic approaches than simple inspection [9]. WSLs are detected in 15.5%-40% of individuals prior to orthodontic therapy while during treatment, WSLs were detected in 30%-70% of patients throughout treatment [3]. According to a meta-analysis, the incidence rate of new carious lesions that occurred during orthodontic treatment was 45.8% in the 14 studies examined for WSLs, with a prevalence rate of 68.4% among individuals undergoing orthodontic treatment. As both the incidence and prevalence of WSLs in orthodontic patients are rather high, this necessitates the attention and care of both the patients and caregivers to effective prevention techniques [18].

Predicting Factors of Post Orthodontic WSL Appearance

Lesions are unique to each patient and their oral environment, varying in size, shape, and location. The amount of brushing, the patient's age, the treatment duration, the tooth type (central incisor or lateral incisor), and the WSL surface area all influenced the betterment of WSL [10]. Several factors were found to be associated with a significant effect on the appearance of WSLs shown in Table 1.

**Table 1:** Factors associated with the appearance of WSLs FOT: fixed orthodontic treatment; DMFT: decayed, missing, filled teeth; WSL: White spot lesion

Factor	Findings
Age	Age at the start of the treatment was significantly associated with WSL development [30]. Another study showed that the age does not play a role in the development of WSL [31]. Each year of age resulted in a 3.1% decrease in WSL surface area over an 8-week period as brushing ability and dexterity may increase with age [10]. Preadolescents ( ≤16 years) were shown to have significantly increased WSL in comparison to adolescents(>16 years) [32].
Gender	Males had significantly higher WSL in comparison to females [32]. Gender was not associated with WSL development [30].
Oral hygiene	Poor oral hygiene significantly affected WSL development [30] Brushing frequency of ≥ 2 times a day was found to be associated with a WSL improvement [10].
Salivary changes during FOT	A rise in salivary flow in all patients was observed as well as considerable drop in salivary pH [33].
DMFT and plaque index	A rise in DMFT index and rise in plaque index was observed in FOT patients [33].
Type of appliance	WSL incidences in subjects treated with the WIN lingual appliance were significantly reduced [25].
Appliance removal time	Greater improvement in WSL appearance was observed with each additional month since appliance removal [10], suggesting that most improvement occurs shortly after appliance removal [10].

Prevention and Treatment of WSL in Orthodontics

Various methods are used during and after orthodontic treatment to prevent enamel demineralization and achieve remineralization. Examples such as “casein phosphopeptide-containing products” and other materials such as anti-bacterials and fluoride-containing products. Chlorhexidine was found to be the most efficient antibacterial material against *Streptococcus mutans*, making it the most used in dentistry [22]. A study reported that combining antibacterial therapy with fluoride administration during fixed orthodontic therapy had a protective effect [34]. To reduce WSL during fixed orthodontic therapy, tooth surface disinfection therapy can be used alongside fluoride and professional mechanical teeth cleaning [22].

Oral Hygiene Control and Frequent Recall Visits

Orthodontic patients exhibit more frequent alterations on the vestibular surface, whereas caries are less likely to develop in untreated patients [13]. Individuals in need of orthodontic treatment should be fully instructed about the need to maintain excellent oral hygiene and should know that orthodontic appliances may hinder full access to the teeth with appliances [22]. Maintaining proper dental hygiene throughout orthodontic treatment needs patient commitment and skill development, which takes time, effort, and motivation [35]. It is more challenging to follow standard oral hygiene procedures in patients receiving orthodontic treatment for plaque removal [36]. The first 6 months are crucial for the patients' WSL development as they adjust to orthodontic therapy's hygiene needs [26].

Antiseptics

Chlorhexidine: Chlorhexidine (CHX) is an antiseptic mouthwash, that inhibits the growth of bacteria, fungi, and viruses that cause various oral disorders. CHX comes in various formulations such as mouthwash, gels, chips, toothpaste, coated brushes, and floss [37]. Mouthwash comes in both alcohol-containing and non-alcohol formulations [38]. For short-term intense plaque management, 0.2% is indicated, while 0.06% can be used daily [37]. It is also given as a 0.12% mouthwash [38]. For mouthwashes, it is recommended to rinse with 10 ml twice a day for 30 seconds [37]. However, it is not recommended for children under the age of 12 without the prior approval of a healthcare professional. It is recommended for short-term usage (two to four weeks) and is permitted for use for 30 days. It is known to reduce plaque accumulation on teeth surfaces [38]. Furthermore, it has been established that its mechanism of action generates antiplaque and antigingivitic effects [39]. However, despite reducing plaque, CHX does not concurrently reduce caries, according to a Cochrane review of eight clinical trials involving adolescents and children [40]. A systematic review of CHX varnishes applied to teeth found no significant evidence that they reduce dental cavities [41]. To prevent caries, a daily mouth rinse with 0.05% sodium fluoride is presently recommended [42]. It may function well in combination with other preventative medications, offering a boosting effect that can be extremely advantageous for high-risk patients [39]. However, using CHX might cause undesirable effects such as dry mouth (xerostomia), changed taste perceptions (hypogeusia), and discolored tongue [37]. Other less common adverse effects include burning sensations (glossodynia), oral mucosal desquamation, and parotid gland enlargement [43]. The primary reason why people avoid using CHX mouthwash is the risk of teeth discoloration [44].

Fluorides

Clinicians and researchers are increasingly interested in conservative techniques for managing WSLs through remineralizing therapy [45]. The use of fluoride in dentistry is one of the most successful preventative health strategies in the history of dental care [46]. Enamel can demineralize in the presence of acid in the oral cavity, leading to the formation of WSLs and ultimately degradation [47].

Fluoride exposure can cause hydroxyapatite crystals to form on the enamel surface. Crystals lose “OH− ions” and replace them with “F− ions”, resulting in “fluorapatite”, which is more resistant to disintegration [47]. This mechanism can occur with small amounts of fluoride (0.01-10 ppm) [46]. Fluoride products are widely used for managing WSLs due to their ease of application, safety, increased enamel contact duration, and patient acceptance [48]. 

Fluoridated Dentifrices: Toothpastes containing sodium fluoride, monofluorophosphate, or stannous fluoride should have a fluoride concentration of at least 1000 ppm [49]. Patients at high risk for WSL benefit from using a toothpaste twice daily containing 5000 ppm of fluoride rather than traditional formulations; however, such toothpastes (Duraphat®, Colgate-Palmolive Company, New York City, USA) cannot be administered for patients under the age of 16 [8]. It has been advocated to use this toothpaste exclusively in the evening [20]. Nonetheless, using fluoride toothpaste alone is ineffective in avoiding WSL in most patients, even with good dental hygiene [50]. As a result, the utilization of supplementary fluoride sources is advised [51].

Fluoridated Mouthwash: Using fluoridated mouthwashes with sodium fluoride daily can significantly reduce carious lesions around and beneath bands. Mouthwashes with antibacterial compounds, such as chlorhexidine, triclosan, or zinc, have been shown to improve caries control [49]. Research suggests that using a daily mouthwash with NaF (Sodium Flouride) (0.05% or 0.2%) can reduce enamel demineralization during fixed orthodontic treatment [8].

Self-applied topical fluorides, such as dentifrices and mouthwashes are commonly used, but their effectiveness is dependent on patient compliance with frequency and dosage. Despite the use of fluoride dentifrices and other standardized measures, new WSLs continue to grow throughout fixed multi-bracketed orthodontic treatment, necessitating alternate preventive treatments [52].

Fluoride varnishes: The development of fluoride varnishes aimed to prolong the contact time, ensure sustained adhesion to the enamel, and thus impede rapid fluoride loss subsequent to application [16]. NaF is present in fluoride varnishes at a concentration of 5% (22,600 ppm). They are applied on dried teeth to ensure adequate enamel penetration [47]. Fluoride varnishes have been shown to effectively prevent and reduce the depth of WSLs in several in vitro and in vivo studies [53-57]. Using a fluoride varnish during orthodontic therapy reduced enamel demineralization by 44.3% [49]. A systematic review and meta-analysis found that fluoride applied topically by a professional (in the form of varnishes or gels) can effectively eliminate the onset of carious lesions in the enamel of permanent or primary teeth [58]. Fluoride varnishes were recommended as an adjunct to active incipient lesions treatment in permanent or primary teeth. It was also found that using fluoride varnishes on a regular basis during fixed orthodontic treatment can protect against WSLs [58]. Fluoride varnishes are safe, despite their high fluoride content [59].

Fluoride Resins: Resin-modified glass ionomer (RMGI) cements have been known for their potential use in caries prevention [47]. Attempts have been made to use fluoride-containing glass ionomer adhesives to bond orthodontic brackets [60]. When comparing the depths of enamel demineralization in proximity to orthodontic bands that are bonded using zinc polycarboxylate, glass ionomer (GI), or RMGI, it was shown that RMGI cement was significantly more effective in preventing enamel demineralization [61]. However, these materials were shown to have low shear-bond strengths in comparison to light-cured composite [62].

Light-Curable Fluoride Varnish: Several studies, including randomized controlled trials [63] and a systematic review [27], suggest that fluoride varnish should be used regularly during fixed orthodontic treatment. Fluoride varnish typically takes many applications to achieve an anti-caries effect [64]. It has been proposed that this frequent application can be significantly decreased with the introduction of light-curable fluoride varnish (LCFV) [65]. LCFV was developed to offer targeted protection for dentin and enamel tooth surfaces [59]. It has proven to be more sustainable and long-lasting than traditional fluoride varnish [66]. RMGI varnishes, such as Vanish™ XT Extended Contact Varnish (3M ESPE, St. Paul, USA) were among the light-curable RMGI varnishes that were introduced to the market in 2009 [59]. Due to the gradual release of fluoride by these compounds, their efficacy may persist for a long duration. Vanish XT varnish is used to treat hypersensitive teeth and seal surfaces with a high caries risk, including recently or partially erupted teeth, orthodontic brackets, acid erosion, and non-cavitated lesions [67]. Vanish XT varnish's glass ionomer formulation improves tooth structure adherence while retaining fluoride [59]. Vanish XT varnish can release calcium and phosphate [68]. Light-curable RMGI varnishes are more effective than fluoride varnishes in preventing enamel demineralization, according to multiple studies [66]. The placement of RMGI cement varnish had a beneficial effect on preventing WSLs during fixed orthodontic therapy [69].

Professional topical fluorides, unlike self-applied fluorides, require healthcare providers to apply high concentrations of fluoride over a short period of time. In order to preserve elevated levels of fluoride on enamel, professional topical fluorides are applied in the form of varnishes, gels, and foams. Their purpose is to impede demineralization and restore mineralization to the impacted regions [70].

Remineralizing Agents

Casein Phosphopeptides Amorphous Calcium Phosphate: Casein phosphopeptide-amorphous calcium phosphate (CPP-ACP) is a bioactive medium with a milk-based base, synthesized from two components: casein phosphopeptides (CPP) and amorphous calcium phosphate (ACP) [71]. In order to facilitate the remineralization of the teeth, casein molecules serve as a vehicle for calcium and phosphate ions [72]. CPP-ACP binds to and maintains the biofilm supersaturated with calcium and phosphate ions, encouraging remineralization [73] by stabilizing existing calcium, phosphate, and fluoride ions in the saliva [15]. However, a systematic review of seven trials found insufficient evidence to support the effectiveness of remineralizing treatments (fluoride and CPP-ACP) for managing post-orthodontic WSLs [53]. However, other systematic reviews and meta-analyses have confirmed CPP-ACP as an effective treatment for WSLs [74, 75]. Also, it was said that CPP-ACP remineralizing paste demonstrated a minor remineralization effect over the three-month evaluation period [76]. It has been reported that, when used with fluoride, this substance can boost the effect of remineralization [16]. Although fluorides and CPP-ACP have demonstrated efficacy in impeding the progression of caries, their esthetic improvement does not meet the standards set by the ICDAS [77]. Although another study showed that CPP-ACP provided a desired and durable esthetic improvement in the management of post-orthodontic white spot lesions (WSLs) in terms of reduction in the area and the color [78].

Sodium Calciumphosphosilicate (Bioactive Glass): When bioactive glass (BAG) comes into contact with saliva, it releases minerals such as sodium, calcium, and phosphorous ions that can remineralize tooth surfaces [79]. The ions form a calcium phosphate layer of hydroxycarbonate apatite (HCA) directly [80]. BAG develops an intimate bond to the tooth structure in less than 2 hours, facilitating apatite production [81, 82]. The newly generated HCA allows growth factors to adhere to the surface [83]. BAG demonstrated its durability after submitting it to a brushing abrasion wear challenge and its ability to penetrate dentinal tubule orifices [84]. It was demonstrated that BAG-containing composite resin could buffer acidic oral conditions by releasing significant quantities of calcium ions. It was additionally shown to produce tooth-like hydroxyapatite crystals. As a result, it is emerging as a viable remineralizing agent that can help prevent WSL formation [85]. The reduced fracture resistance and limited mechanical strength could be considered a minor drawback of BAG [86]. Brown et al. examined four BAG-Bond materials, two of which contained fluoride. It was found that each BAG-Bond induced a substantial discharge of calcium and phosphate, in addition to a notable alteration in pH [87].

Nanohydroxy-Appetite (nano-HAP): The translucency and white appearance of enamel have been attributed to hydroxyapatite (HAP), which makes up most of the inorganic component of the dental hard tissue. With the development of nanotechnology, the use of nanohydroxyapatite (nano-HAP) in dentistry has attracted interest due to its superior mechanical, physical, and chemical qualities. It is among the most biocompatible and bioactive dental materials [88]. The biomimetic nano-HAP protects teeth by creating a new layer of synthetic enamel around them, rather than hardening the existing layer with fluoride [89]. Nano-HAP has unique features such as increased solubility, higher surface energy, and superior biocompatibility [90]. A study examined the remineralizing ability of nano-HAP under a pH cycling model. Also, the same study stated that the addition of nano-HAP to dentifrices and mouthwashes showed to remineralize artificial demineralized lesions [91]. Premolars and molars exhibited remineralization of initial approximal enamel and dentine subsurface lesions in response to ozone therapy and nano-HAP gel [92]. Another study conducted a comparison of the extent of microleakage beneath orthodontic bands fabricated with luting glass-ionomer cement and nano-HAP at various concentrations (0%, 5%, 10%, and 15% by weight). A significant effect of nano-HAP was observed in the reduction of microleakage around orthodontic bands, particularly when 15% nano-HAP-enriched glass-ionomer cement was applied [93]. Even though nanotechnology is already an integral part of our daily lives, the safety of nanomaterials has been the subject of considerable debate [88]. As it may be associated with a multitude of adverse biological effects that manifest both locally and systemically [88].

Self-Assembling Peptides

An emerging strategy for masking and reversing white spot lesions is using the self-assembling peptide P11-4, which serves as a biomimetic approach for enamel regeneration [94]. Damaged tissues are replaced with tissues that are biologically similar; this represents a transition from reparative to regenerative dentistry [95]. Under physicochemical conditions, upon application of the solution containing P11-4, it transforms into ribbons and tapes within seconds, and fibrils and fibers within the subsequent 24 hours. Self-assembling peptide fibers that comprise the 3D Self-Assembling Peptide Matrix (SAPM) have the capability to proliferate into a substantial length of peptide as it diffuses into the lesion [94]. It is hypothesized that within the lesion, it spontaneously self-assembles into three-dimensional polymers composed of self-designed β-sheet aggregates. Optimal attachment of calcium and phosphate from saliva is anticipated to occur in this manner; this process is referred to as "salivary-driven remineralization". Ultimately, de novo hydroxyapatite generation [96]. Wierichs et al. reported the disadvantages associated with the self-assembling peptide technique. It was determined that in oral environments, the nematic form of a self-assembling peptide experiences flocculation, characterized by alternating cycles of demineralization and remineralization, which cause pH fluctuations. The self-assembling peptide in this flocculated state is comparatively inert and has the potential to impede the remineralization process. In addition, they stated that the incorporation of these flocculates into the enamel during the remineralization process affects calcium, phosphate, and fluoride ion diffusion to the enamel surface. After demineralization, the availability of fluoride ions is consequently diminished [97]. A recent systematic review concluded that the self-assembling peptide P11-4 group exhibited superior biomimetic remineralization in both in-vitro and in-vivo studies compared to other remineralizing agents. Nevertheless, to validate its suitability for clinical application, long-term studies are required [94].

Laser Application

Lasers have been regarded as potentially effective in the prevention of dental caries, as it was stated that heating the enamel surface inhibits caries as heating the enamel surface alters its organic and inorganic components [98]. Laser has been reported to improve enamel resistance against decay [99]. Stern and Soggnaes were the first to show that laser irradiation of the enamel surface increased tooth acid resistance in 1972 [100]. Different types of lasers have been mentioned in the dental literature including argon, CO2, Er:YAG, Er:Cr:YSGG, and Er:YLF lasers, and Nd-YAG [98].

Bleaching

Bleaching methods have limited aesthetic results and may cause dental sensitivity and diminished enamel microhardness [101]. A study found that bleaching incipient enamel caries with 10% carbamide peroxide can conceal WSLs without affecting the enamel's chemical or mechanical properties. Additionally, CPP-ACP was used as an adjunct treatment to promote mineral gain in the subsurface lesion [10]. In an in vitro study, Khoroushi et al. found that by integrating three biomaterials such as nano-amorphous calcium phosphate, nano-BAG, and nano-hydroxyapatite into bleaching materials, adverse effects can be mitigated and irreversible enamel surface alterations prevented [102]. Such a modality is advised for individuals with good oral hygiene to conceal WSL when spontaneous remineralization is incomplete [103].

Microabrasion

Microabrasion can improve the appearance of teeth by removing the outer, damaged layer of enamel, thereby promoting remineralization of the demineralized underlying enamel [104]. Microabrasion uses 6.6% hydrochloric acid and 20-160-µm-sized silicon carbide microparticles to eliminate superficial lesion areas through chemical erosion and mechanical abrasion [105]. Microabrasion esthetically enhances WSLs by removing the superficial layer, creating a smoother and glossier surface [106]. It was demonstrated that microabrasion increased the aesthetics of WSLs and showed adequate durability after 12 months [107]. This procedure is effective for treating post-orthodontic WSLs but requires a lesion depth of less than 0.2 mm [20] and may need bleaching [103]. The microabrasion technique, regardless of the type of abrasive technique used i.e. 18%, 35%, or 6.6% hydrochloric acid, leads to increased enamel surface roughness [108, 109]. Also, enamel microabrasion leads to decreased microhardness [110]. However, microabrasion is considered a safe, conservative, atraumatic technique for removing superficial enamel lesions [111].

Resin Infiltration

Resin infiltration involves obturating the microporous enamel portions of noncavitated early carious lesions with low-viscosity light-cured resins, preventing further caries progression [112]. Resin infiltration can hide WSLs, in addition to preventing caries [113]. The infiltrant's refractive index (1.52) is like that of enamel/apatite (1.62), compared to water's (1.33) and air's (1.00) indices. As infiltration increases, light scattering decreases [97]. The resin infiltration approach involves improving enamel porosity, by applying 15% hydrochloric acid to the surface layer. Then, using a low-viscosity resin - dimethacrylate-based triethylene glycol resin - to infiltrate hypocalcified or demineralized enamel microporosities up to 58±37 μm in depth [114]. The proposition of employing low-viscosity resins to fill the porous structure rather than entirely eliminating the initial carious lesions, would not only diminish the micropore structure but also provide mechanical reinforcement to the enamel tissue [115]. Resin infiltration and microabrasion are invasive and microinvasive techniques, respectively, that can effectively mask more severe and persistent cases of post-orthodontic WSLs. However, both approaches are technique-dependent; the former typically requires multiple repetitions, whereas the latter typically only requires one appointment [116].

Table 2 shows the advantages and disadvantages of different useful in the prevention and management of orthodontically induced WSLs. 

**Table 2:** Advantages and disadvantages of different treatment modalities of orthodontically induced WSLs CPP-ACP: Casein phosphopeptide-amorphous calcium phosphate; WSL: White spot lesion

Technique	Advantage	Disadvantage
Prevention		
Chlorhexidine	Very effective in reducing *Streptococcus mutans*	Altered taste and teeth discoloration
Fluorides	Enamel remineralization	Dentifrices and mouthwash require compliance. Varnishes and gels require repeated applications.
CPP-ACP	Inhibit demineralization	Minor remineralization effect Contraindication: Milk allergies
Bioactive glass	Enamel remineralization by facilitating apatite production and durability	Low mechanical strength and decreased fracture resistance
Nanohydroxy appetite	Remineralizing early enamel caries	Safety and lack of long-term studies
Self-assembling peptide P_11-4_	Superior biomimetic remineralization	May impede the remineralization process in its flocculated state
Treatment		
Laser	Improve enamel resistance against acid attacks	Effectiveness in preventing WSLs during orthodontic treatment Requires special training
Bleaching	Concealment of WSLs	Dental sensitivity and diminished enamel microhardness
Microabrasion	Esthetically enhances WSLs by removing the superficial layer	-Only used with superficial lesions restricted to enamel -Increases the surface roughness of the of the enamel
Resin Infiltration	Camouflages the WSL	The durability of the results due to staining and aging of the material

Sequence of WSL Therapeutic Management

**Figure 2:**
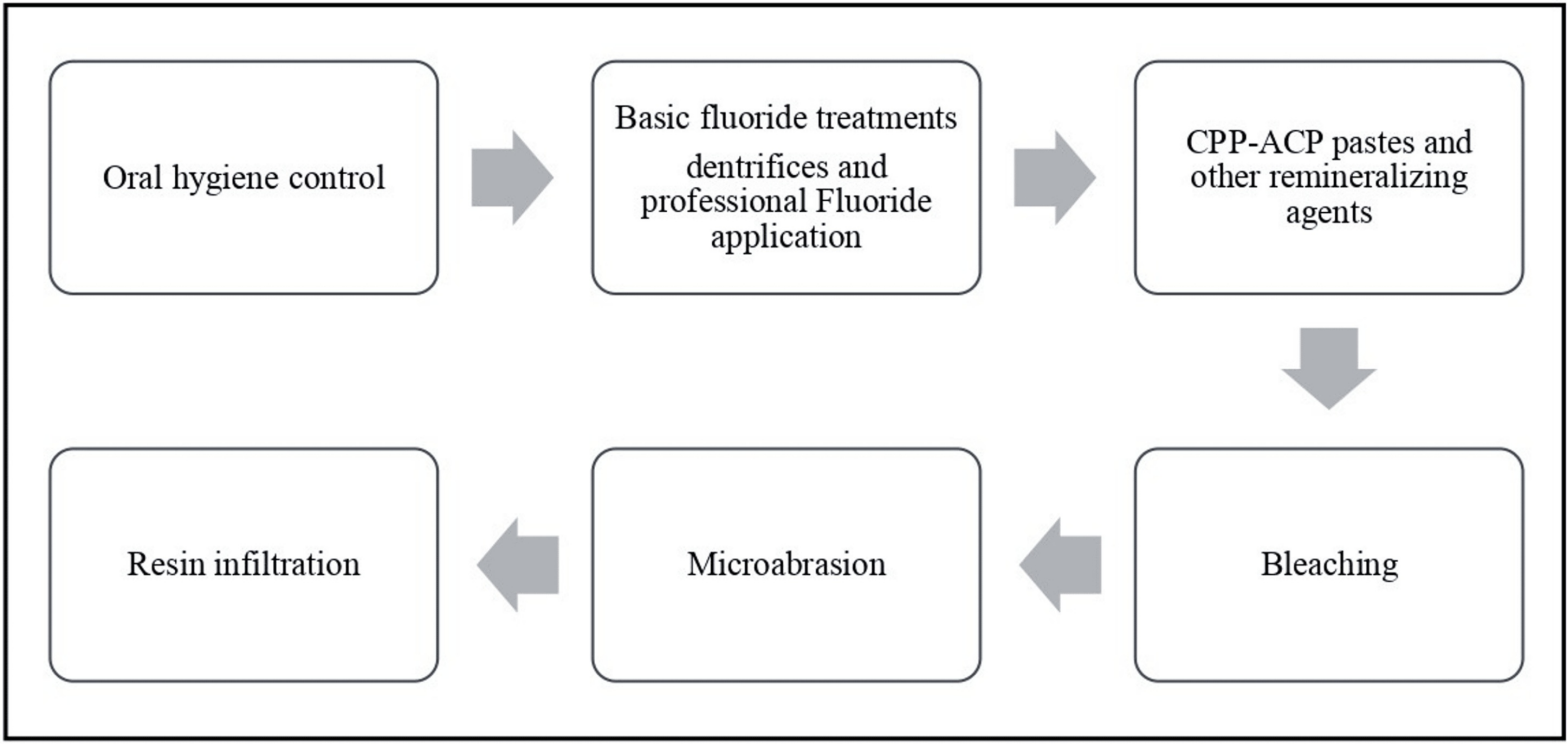
Sequential treatment of orthodontically induced WSL CPP-ACP: Casein phosphopeptide-amorphous calcium phosphate; WSL: white spot lesion

The treatment of post-orthodontic demineralization should use a top-down strategy [15]. Thereby, beginning with the least invasive and most preventative procedures as shown in (Figure 2). Prevention and remineralization of demineralized enamel should be the first therapeutic approach. If this fails to occur, it signifies that the deterioration has progressed to the more profound levels of the enamel, remaining solely on the mineral-rich enamel surface. Under such conditions, resin infiltration or other therapeutic alternatives will be necessary to seal the microporosities.

Limitations

This review summarizes the common treatment modalities of WSL. However, it has some limitations due to the small number of studies reviewed. This review adds to existing knowledge and supports further research on the topic. Additionally, review articles are susceptible to publication bias due to the higher likelihood of publishing positive or significant findings. Underrepresentation of studies with adverse outcomes can lead to biased review conclusions.

Clinical implications

Despite advancements in orthodontic techniques, materials, and diagnostics, dentists still encounter demineralization around brackets. Although preventative therapies exist, they cannot replace patient compliance and constant home care as poor oral hygiene reduces the effectiveness of preventative actions. Orthodontic practitioners should motivate their patients to perform the necessary oral hygiene routine to help control WSLs, with the use of fluorides and other remineralizing agents CPP-ACP. Long-term research is needed to evaluate the effectiveness of various treatment approaches for post-orthodontic WSL.

## Conclusions

Enamel decalcification around fixed orthodontic appliances is a common consequence of orthodontic therapy, as it raises the risk of carious demineralization by increasing the area occupied by cariogenic bacteria and complicating plaque removal. To control these lesions, patients are educated and motivated to practice proper oral hygiene and dietary measures. A clear and in-depth knowledge of the mechanism underlying the formation and progression of subsurface lesions is needed, as well as the potential uses and limitations of the current treatment modalities and their clinical applications, to target individuals at risk for caries with preventive measures and minimally invasive treatment as necessary.
